# Resveratrol Inhibits Cancer Cell Metabolism by Down Regulating Pyruvate Kinase M2 via Inhibition of Mammalian Target of Rapamycin

**DOI:** 10.1371/journal.pone.0036764

**Published:** 2012-05-04

**Authors:** Mohd Askandar Iqbal, Rameshwar N. K. Bamezai

**Affiliations:** National Centre of Applied Human Genetics, School of Life Sciences, Jawaharlal Nehru University, New Delhi, India; University of Nebraska Medical Center, United States of America

## Abstract

Metabolism of cancer cells with pyruvate kinase M2 (PKM2) at its centre stage has assumed a prime significance in cancer research in recent times. Cancer cell metabolism, characterized by enhanced glucose uptake, production of lactate and anabolism is considered an ideal target for therapeutic interventions. Expression of PKM2 switches metabolism in favor of cancer cells, therefore, the present study was designed to investigate the hitherto unknown effect of resveratrol, a phytoalexin, on PKM2 expression and resultant implications on cancer metabolism. We observed that resveratrol down-regulated PKM2 expression by inhibiting mTOR signaling and suppressed cancer metabolism, adjudged by decreased glucose uptake, lactate production (aerobic glycolysis) and reduced anabolism (macromolecule synthesis) in various cancer cell lines. A contingent decrease in intracellular levels of ribose-5-phosphate (R5P), a critical intermediate of pentose phosphate pathway, accounted for a reduced anabolism. Consequently, the state of suppressed cancer metabolism resulted in decreased cellular proliferation. Interestingly, shRNA-mediated silencing of PKM2 inhibited glucose uptake and lactate production, providing evidence for the critical role of PKM2 and its mediation in the observed effects of resveratrol on cancer metabolism. Further, an over-expression of PKM2 abolished the observed effects of resveratrol, signifying the role of PKM2 downregulation as a critical function of resveratrol. The study reports a novel PKM2-mediated effect of resveratrol on cancer metabolism and provides a new dimension to its therapeutic potential.

## Introduction

Cancer cells rely on the process of metabolic transformation to sustain proliferation. These cells bypass mitochondrial oxidative phosphorylation, which channelizes glucose for ATP production (catabolism), and instead utilize glucose for the macromolecule synthesis (anabolism) for daughter cells [1]. Cancer cells also convert most of pyruvate (terminal product of glycolysis) into lactate, through largely unknown mechanism; and thereby prevent its entry into mitochondria [2]. Increased glucose uptake and lactate production are hallmarks of cancer metabolism (Warburg effect or Aerobic glycolysis) [2], providing cancer cells an advantage to grow even in regions with very low oxygen concentration [1], [2]. Growth factor signaling mediated by mammalian target of rapamycin (mTOR) drives metabolism of cancer cells by regulating expression of key enzymes in metabolic pathways [3]. Notably, mutations causing hyper-activation of mTOR are common in cancers [4], [5].

Pyruvate kinase M2 (PKM2) is shown to be vital for cancer metabolism and critical to tumor growth [6]. Out of the four isoforms of pyruvate kinase L, R, M1 and M2 [7], [8], proliferating embryonic and tumor cells predominantly express M2 [1]. This isoform switch is a pre-condition for aerobic glycolysis to occur [9].

PKM2, an allosteric isoform of pyruvate kinase, catalyzes the last step in glycolysis i.e. conversion of phosphoenol-pyruvate to pyruvate [10], [11]. Since PKM2 has a lower activity compared to the constitutively active isoforms [1], its suppression in activity causes an accumulation of upstream glycolytic intermediates. A consequent result is increased availability of glycolytic intermediates for biosynthetic pathways like pentose phosphate pathway (PPP), which accelerates macromolecule biosynthesis (Ribose-5-phosphate; R5P) and tumor growth [1]. R5P is an important intermediate of pentose phosphate pathway and serves as a precursor for synthesis of macromolecules [12]. The expression of PKM2 in tumors is high enough to be exploited as a marker for cancer prognosis [13], [14], [15]. Our laboratory has reported natural mutations in PKM2 associated with increased cellular proliferation and polyploidy [16]; emphasizing the role of PKM2 in cellular proliferation. Hence, screening drugs which effectively inhibit PKM2 expression and prevent metabolic transformation becomes pertinent [17].

Resveratrol (3, 4′, 5-trihydroxystilbene) is a phytoalexin, present in the skin of red grapes and other fruits [18]. Resveratrol is reported to exert antitumor activities at various stages of tumor initiation, promotion and progression [19]. This is corroborated by reports of chemo-preventive effect of resveratrol in various cancer cell lines like, HeLa, A549 and MCF-7 [20], [21], [22]. Various mechanisms for anti-proliferative action of resveratrol have been proposed including increase in tumor suppressor proteins like, p53 [22], BRCA 1&2 [23], phosphorylation of Rb protein and transcription factors like NF-kB and AP-1 [24]. In a recent report, resveratrol was shown to modulate cellular proliferation via SIRT1-dependent AMPK activation [25]. Resveratrol is reported to inhibit PI3K pathway and affects glycolysis to cause cell cycle arrest in B cell lymphomas [26]. Resveratrol-mediated inhibition of mTOR is also known [27]. However, these findings do not explain the role of resveratrol in metabolic transformation, especially when coupled to PKM2 expression. We report in this study, the effect of resveratrol on PKM2 expression with consequent implications on cancer metabolism. For the first time, our results demonstrate novel PKM2-mediated effect of resveratrol on cancer metabolism that corroborates with its therapeutic potential.

## Materials and Methods

### A) Cell culture, drug treatment, PKM2 knockdown, transfections and cellular proliferation studies

HeLa, HepG2 and MCF-7 cell lines were procured from the National Centre for Cell Science, Pune, India. All the cell lines were maintained in high glucose dulbecco's modified eagle's medium (DMEM; Sigma, MO, USA) with 10% fetal bovine serum (FBS) (Biowest, France), 1% penicillin/streptomycin (Sigma) at 37°C and 5% CO_2_ in a humidified atmosphere. Cells were grown in monolayer and passaged routinely 2–3 times a week. For drug treatment, resveratrol (Sigma) and rapamycin (Sigma) were dissolved in ethanol and dimethyl sulfoxide (DMSO) respectively; aliquots were stored at −80°C. Cells were seeded in triplicate at a density of 0.1–0.2 million/well in six well plates. Prior to drug treatment, cells were incubated for 24 hours and thereafter replaced with resveratrol containing media, followed by 48 hours incubation. Ethanol and DMSO-treated cells were used as a mock control. Lentiviral pGIPZ (Open Biosystems, USA) was used for shRNA-mediated silencing of PKM2. For PKM2 over-expression studies, pCDNA-myc and pCDNA-myc-PKM2 tagged vectors were used. Lipofectamine LTX (Invitrogen, USA) was used as a transfection reagent. For proliferation studies, cells were counted, seeded and then trypsinized at different time points followed by repeat counting to assess proliferation rate.

### B) RNA isolation, cDNA preparation and real time analysis

Total RNA was extracted from cell lines using TRIzol (Sigma) according to manufacturer's protocol. RNA quality was analyzed by A_260_/A_280_ absorbance equilibrium and by electrophoresis on 1.2% agarose formaldehyde gel. 1–2 µg of total RNA was reverse transcribed into single stranded DNA using cDNA preparation kit (Applied Biosystems, USA). Commercially available Taqman gene expression assay (Applied Biosystems) with part number Hs00987621_g1 was used for quantitating mRNA levels of PKM2. Actin was used as endogenous control (Part No. 4333762F, Applied Biosystems, USA). Primer probes were chosen to avoid contaminating genomic DNA amplification. Real time PCR was carried out on ABI Prism 7000 Sequence Detection System (Applied Biosystems, USA). ΔΔC_t_ (Cycle threshold) method of relative quantification was used to calculate fold change in gene expression by SDS 1.1 RQ software (Applied Biosystems, USA).

### C) Cell lysate preparation, Protein estimation and Western Blotting

Whole cell lysate was prepared by incubating cells on ice for 30 minutes in buffer containing 50 mM Tris pH 7.2, 150 mM NaCl, 0.5% sodium deoxycholate, 10% glycerol, 1% Triton X-100, 0.1% SDS, 1 mM dithiothreitol (DTT), 1 mM phenylmethylsulfonyl fluoride (PMSF), 5 mM sodium fluoride (NaF), 1 mM sodium vanadate (NaV), phosphatase inhibitor cocktail (Sigma), 4 µg/ml aprotinin, 4 µg/ml leupeptin and 4 µg/ml pepstatin (Sigma). The lysate was centrifuged at 12000 rpm in a cooling centrifuge (CM 12, Remi, India) for 15 minutes and supernatant was collected in ice-chilled fresh tubes. Protein concentration was estimated using Pierce BCA (bicinchoninic acid) protein assays as per the manufacturer protocol. Proteins were separated on 10% SDS-PAGE, transferred to nitrocellulose membrane (mdi, USA) overnight at 4°C (wet transfer), and probed with primary antibodies. Membrane was incubated with appropriate secondary antibody for 1 hour at room temperature and proteins were detected using enhanced chemiluminescence kit from Thermo Scientific, USA. Primary antibodies used were: anti-PKM2, anti-myc, anti-p-p70S6K, anti-p70S6K and anti-β-actin (Cell signaling Technology, USA).

### D) Glucose uptake and lactate production assay

Media was collected from wells and glucose uptake was analyzed using glucose (Hexokinase) assay kit (Sigma) as per the manufacturer's instructions. Lactate production was analyzed using lactate assay (BioVision, USA) by following manufacturer's specifications. All the measurements were normalized to cell numbers.

### E) Metabolite extraction and estimation by LC-MS

Metabolite extract was prepared from 5 million cells using 0.5 ml of 90% chilled ethanol containing 0.5% formic acid and centrifuged for 30 minutes at high speed in a refrigerated centrifuge. Thereafter, supernatant was dried using nitrogen flow and reconstituted in 0.2 ml MilliQ water. LC-MS was performed as per specifications described previously [28]. Percent change in signal (intensity) was calculated taking mock treated control as reference; negative change in signal depicted the decrease in the levels of metabolites.

### F) Statistical analysis

All experiments were repeated 3 times and are expressed as mean ± SE. *p* values were calculated using *student's t test* and *p<0.05* was considered significant.

## Results

### Resveratrol decreases PKM2 mRNA and protein expression via mTOR inhibition

**Figure 1:**
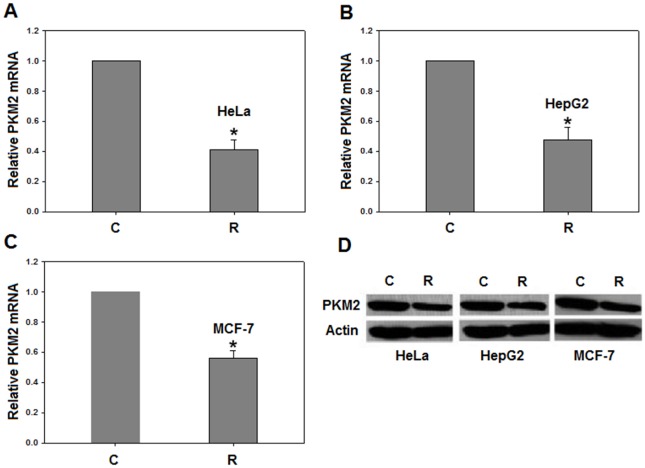
Resveratrol decreases PKM2 expression. Resveratrol treatment decreases PKM2 mRNA in (A), HeLa; (B), HepG2 and (C), MCF-7; by approximately two-folds. β-actin was taken as endogenous control and normalized to PKM2 mRNA. Relative quantification analysis was done using SDS 1.1 RQ software. Western blot showing decreased PKM2 protein in all the cell lines studied on treatment with 50 µM resveratrol (D). *C- Mock treated control and R- resveratrol treated.* All experiments were repeated 3 times and data is expressed as mean ± SE. *p<0.05.

PKM2 mRNA and protein levels were analyzed, using real time PCR and Western blotting, in resveratrol treated HeLa, HepG2 and MCF-7 cells. We chose 50 µM resveratrol for 48 hour exposure since this treatment increases the percentage of cells blocked in S phase [20]. It is also established that PKM2 expression is at maximum in S phase [29]. Interestingly, resveratrol down-regulated PKM2 mRNA and protein by ∼2 fold in all cell lines studied (Figure 1A–D). These results provide first evidence of PKM2 expression being affected by resveratrol.

**Figure 2:**
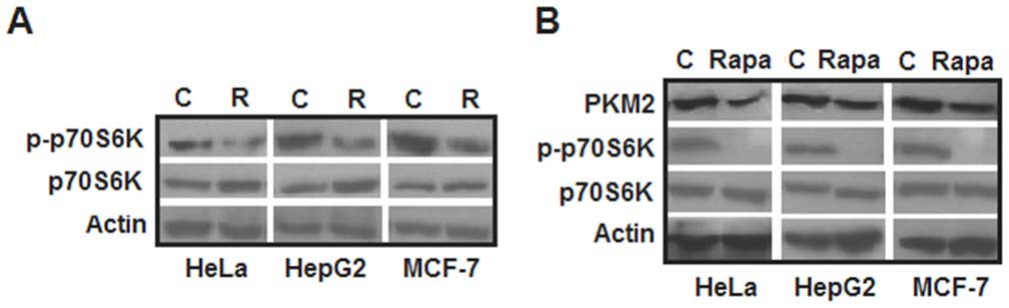
Western blots showing the effect of resveratrol on mTOR signaling and rapamycin (mTOR inhibitor) on PKM2 expression. Resveratrol (50 µM) inhibited mTOR signaling in all the three cell lines studied as evident from decreased phosphorylation of p-p70S6K upon resveratrol treatment (A); *C- Mock treated control and R- resveratrol treated*. mTOR inhibition by 20 nM rapamycin reduced PKM2 expression in all experimental cell lines (B); *C- Mock treated control and Rapa- 20 nM Rapamycin*.

In order to investigate how resveratrol is down regulating PKM2 expression, we looked into the mTOR signaling pathway, which is frequently dysregulated in cancers [30], [31], [32], [33]. mTOR pathway is also known to regulate expression of various glycolytic enzymes including PKM2 [3], [34]. Upon resveratrol treatment, we observed inhibition of mTOR signaling in all the cell lines studied (Figure 2A). Inhibition of mTOR by its well-known inhibitor rapamycin (20 nM for 24 hours) also reduced PKM2 expression (Figure 2B). These results showed that resveratrol down-regulated PKM2 expression by mTOR inhibition.

### Decreased glucose uptake and lactate production on resveratrol treatment

**Figure 3:**
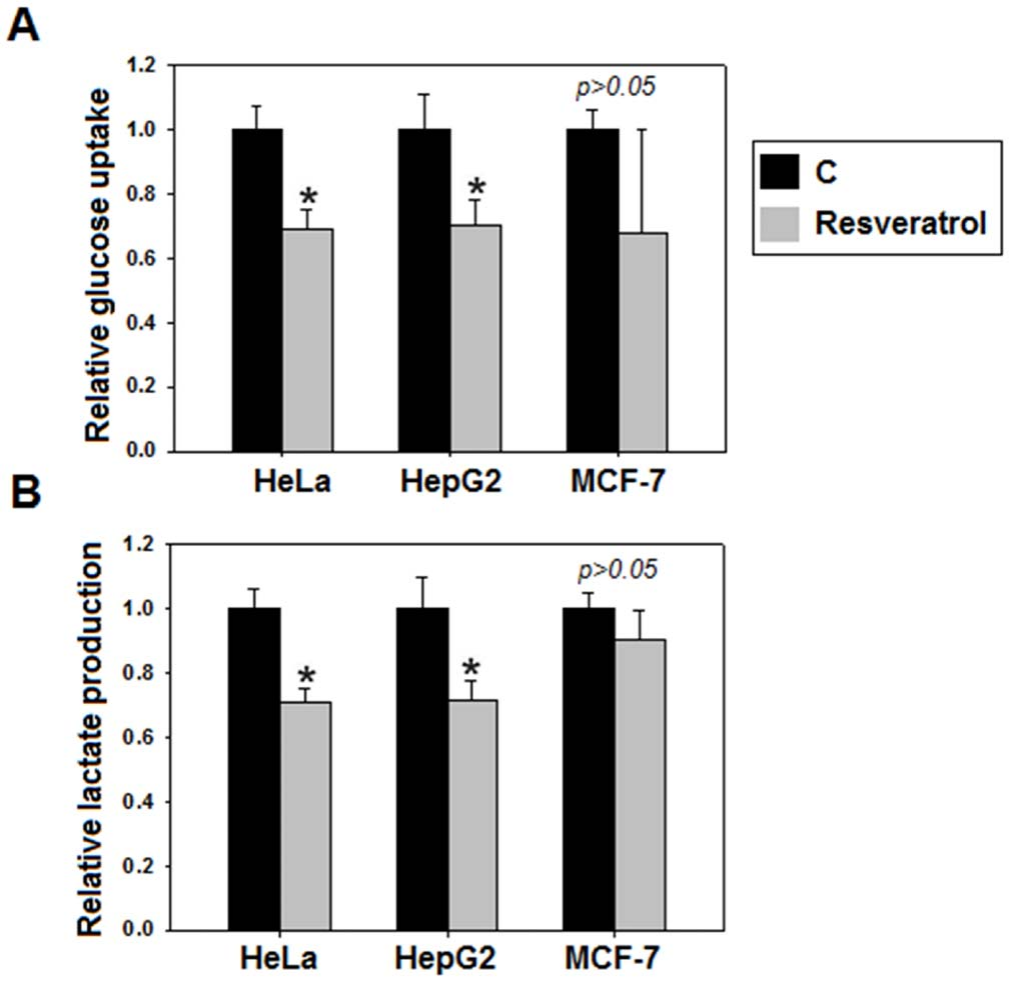
Resveratrol inhibits aerobic glycolysis (glucose uptake and lactate production characteristic of cancer metabolism). A significant decrease in glucose uptake (A) and lactate production (B), was observed in HeLa and HepG2 (*p<0.05), following 48 hours resveratrol treatment. However, in MCF-7 aerobic glycolysis did not show significant signs of inhibition (*p>0.05*). All experiments were repeated 3 times and data is expressed as mean ± SE.

Cancer cells require a large and continuous supply of glucose for anabolic processes. The lactate production by cancer cells prevents the entry of pyruvate into mitochondria [2]. Thus, increased glucose uptake and lactate production are hallmarks of cancer metabolism. Effect of resveratrol on cancer metabolism was studied by assessing the changes in glucose uptake and lactate production. Interestingly, we observed a substantial decrease in glucose uptake and lactate production on resveratrol treatment (Figure 3A–B), demonstrating that resveratrol inhibits aerobic glycolysis, which is suggestive of suppressed cancer metabolism.

### Silencing of PKM2 results in decreased aerobic glycolysis and cellular proliferation

**Figure 4:**
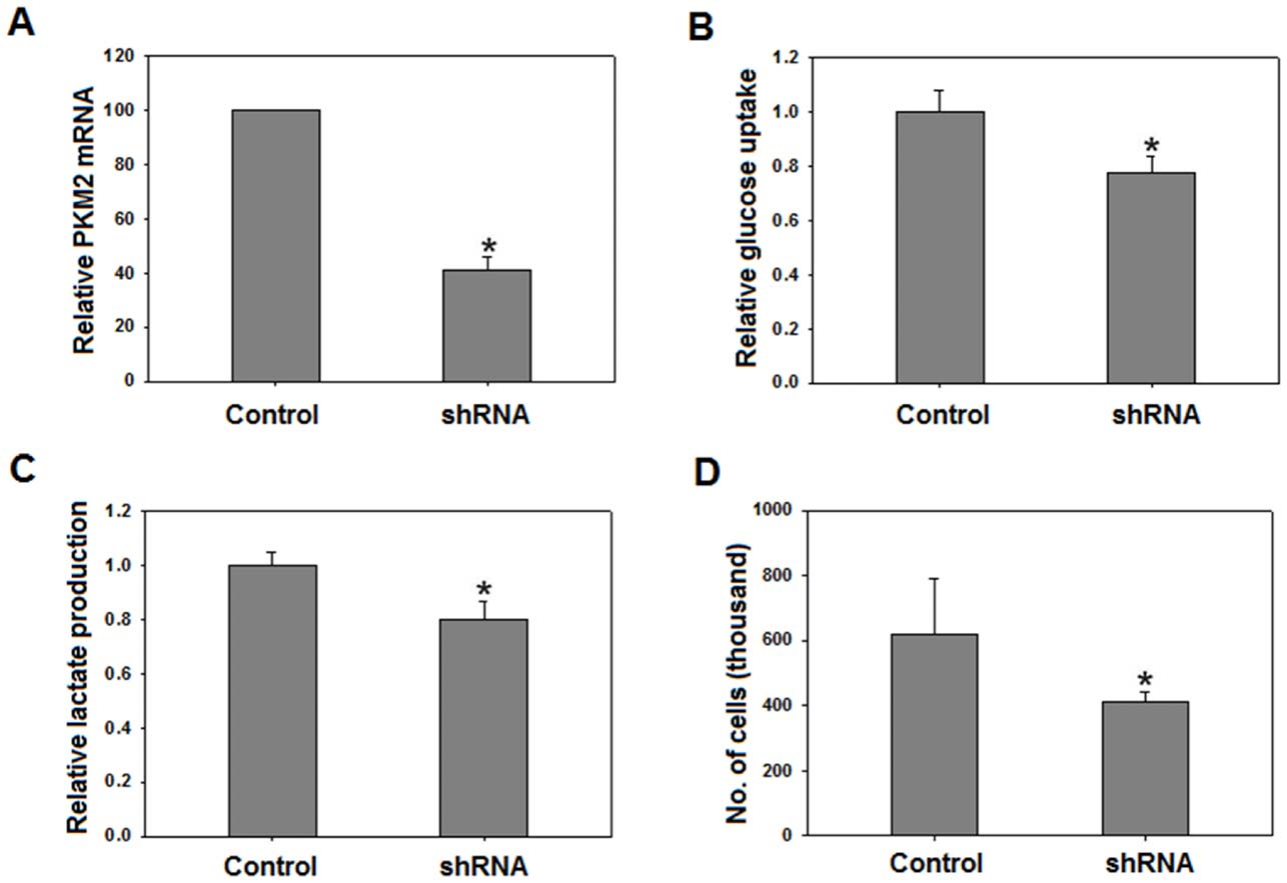
PKM2 knockdown reduced aerobic glycolysis. PKM2 was knocked down (nearly 60% – using lentiviral pGIPZ vector containing PKM2 shRNA) as analyzed by: real time PCR (A), decreased glucose uptake (B), lactate production (C), and cellular proliferation (D) following knock down. All experiments were repeated 3 times and data is expressed as mean ± SE. *p<0.05.

We postulated that the observed decrease in aerobic glycolysis was partly due to decreased PKM2 expression. To test this assumption, we silenced PKM2 using shRNA. PKM2 knock down achieved (∼60–70%) was assessed by real time PCR (Figure 4A). Following knockdown, glucose uptake and lactate production was measured. Cells with silenced PKM2 showed a significant reduction in glucose uptake and lactate production, suggesting that PKM2 is required for aerobic glycolysis (Figure 4B–C). Decreased aerobic glycolysis resulted in inhibition of cellular proliferation (Figure 4D). These results confirmed that PKM2 is critical for cancer metabolism and cellular proliferation. Results affirmed that resveratrol inhibits cancer metabolism via PKM2.

### Resveratrol decreases anabolism in cancer cells

**Figure 5:**
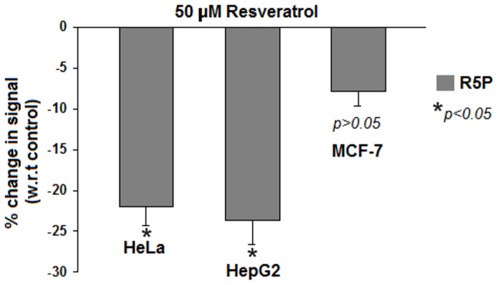
Resveratrol decreased intracellular levels of R5P. R5P is a pentose phosphate pathway intermediate, an important marker for anabolism (nucleotide biosynthesis). Approximately 20–25% decrease in intracellular levels of R5P in HeLa and HepG2 was observed upon 50 µM resveratrol treatment. However, there was an insignificant decrease of around 5% in MCF-7 cells (see text). Results indicated inhibited anabolism following resveratrol treatment. All experiments were repeated 3 times. Values are expressed as mean ± SE. *p<0.05.

Ribose-5-phosphate, an anabolic marker, is required for macromolecule synthesis. It is an intermediate in pentose phosphate pathway [12]. To examine if resveratrol negatively affects anabolism in cancer cells, we measured intracellular levels of R5P in cell lines treated with resveratrol. Metabolites were extracted from treated cell lines followed by LC-MS analysis. Interestingly, reduction of ∼20–25% was found in R5P levels in resveratrol treated cells, suggesting inhibited anabolism (Figure 5). MCF 7 cells showed a relatively small decrease in R5P levels indicating a weakly suppressed anabolism.

### Suppressed cancer metabolism inhibits cellular proliferation

**Figure 6:**
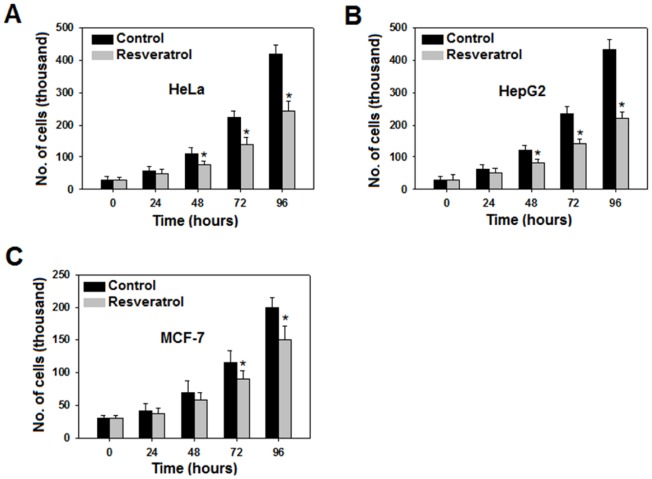
Cellular proliferation retarded as a result of suppressed cancer metabolism. To examine how inhibition of cancer metabolism affects proliferation of cell lines, we trypsinized and counted cells at 0, 24, 48, 72 and 96 hours. There was a decrease in proliferation rate in all the three cell lines studied with prominent decrease in HeLa (A), HepG2 (B) and least prominent in MCF -7 (C). All experiments were repeated 3 times and data is expressed as mean ± SE. *p<0.05.

To study how the proliferation rate of various cell lines is affected following resveratrol treatment, we treated HeLa, HepG2 and MCF7 cells with 50 µM resveratrol for 0, 24, 48, 72 and 96 hours and counted the cells. Effect was prominent in HeLa and HepG2 but was feeble in MCF7 (Figure 6A–C) cells. Nevertheless, resveratrol inhibited cellular proliferation, suggesting that suppression of cancer metabolism retarded cell proliferation as well.

### Over-expression of PKM2 reverses the effects of resveratrol

**Figure 7:**
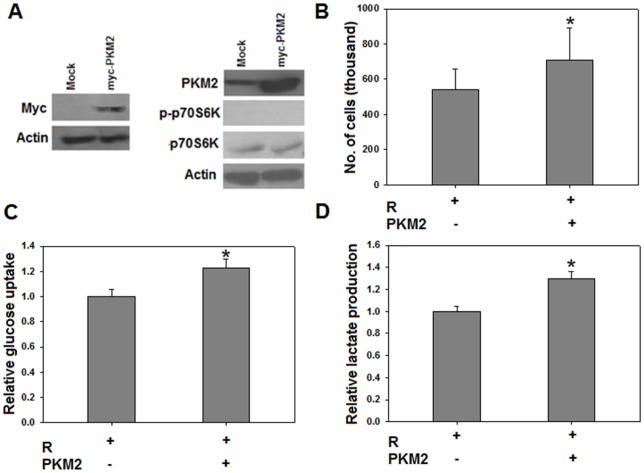
PKM2 over-expression abolished the effects of resveratrol. Over-expression of PKM2 using myc tagged pCDNA vector (see text) was confirmed using anti myc and anti PKM2 antibodies (A). Increased cellular proliferation in PKM2 transfected cells, compared to mock transfected cells, in continuous presence of inhibitory concentrations of resveratrol (B). Glucose uptake (C) and lactate production (D) was substantially enhanced in PKM2 transfected cells indicating that PKM2 over-expression abrogates effects of resveratrol on cancer metabolism and cellular proliferation. These results further validate PKM2 as critical target of resveratrol. All experiments were repeated 3 times and data expressed as mean ± SE. *p<0.05. *PKM2 – indicates mock transfected cells whereas PKM2+indicates PKM2 transfected cells*.

To confirm our findings of resveratrol in cancer metabolism and hence proliferation by targeting PKM2, we transiently over-expressed PKM2 in HeLa cells exposed to media containing 50 µM resveratrol. Cells were transfected either with pCDNA-myc (Mock transfection) or with pCDNA-myc-PKM2 (PKM2 transfection). After 48 hours of transfection (and simultaneous resveratrol treatment), cells were counted, harvested and lysed for protein extraction. Transfection was confirmed using anti-myc and anti-PKM2 antibodies (Figure 7A); so was the inhibition of mTOR (Figure 7A). As expected, over-expression of PKM2 resulted in increased cellular proliferation, compared to mock transfected cells even in the presence of 50 µM resveratrol (Figure 7B). Moreover, PKM2 over-expression caused augmented glucose uptake and lactate production (Figure 7C and D) in HeLa cells exposed to resveratrol; counteracting the negative effects of resveratrol. These results further substantiated that PKM2 is a critical target of resveratrol and its expression determines cancer metabolism and consequently the cellular proliferation.

## Discussion

Metabolic transformation is considered as an indispensable change acquired by cancer cells to thrive. Research into the metabolic addictions of cancer cells has accelerated in recent years. Metabolic phenotype of cancer cells is believed to engage enzymes as suitable targets for anticancer strategies. Drugs that inhibit metabolism of cancer cells by targeting a variety of molecules (including enzymes) directly or indirectly, are under clinical trials [17]. It is therefore pertinent to screen drugs with a potential to target critical molecules involved in metabolic transformation.

Resveratrol, though known for its anticancer properties, has never been associated with crucial metabolic enzymes like PKM2. Its relation with cancer metabolism is poorly understood. We have for the first time shown resveratrol affects PKM2 status, thus inhibiting cancer metabolism.

It has been reported earlier that resveratrol affects glucose metabolism in ovarian cancer cells [35]. Faber *et al* have shown decreased expression of some glycolytic enzymes on resveratrol treatment [27], but their observations do not indicate resveratrol altering cancer metabolism by affecting the status of critical molecules like PKM2. Since PKM2 has recently been identified as a key player in promoting cancer metabolism and tumor growth [6], it was imperative to investigate if resveratrol could alter PKM2 to affect metabolism of cancer cells.

PKM2, because of its position in glycolysis, promotes biosynthetic pathways (e.g. PPP) required for macromolecular synthesis [2], [6], [12]; and therefore its expression is highest in S (synthesis) phase of cell cycle [29]. We have shown that down regulation of PKM2, by resveratrol, inhibits biosynthetic pathways required for synthesis of macromolecules (Figure 5). These results possibly explain the observed accumulation of cells in G0/G1 phase on resveratrol treatment [26]; and suggest how resveratrol inhibits cancer metabolism by targeting PKM2.

The decrease in glucose uptake and lactate production (aerobic glycolysis) by resveratrol widens its chemopreventive spectrum and emphasizes its therapeutic value since aerobic glycolysis provides a life-line for cancer cells. By demonstrating that cancer metabolism inhibitory effects of resveratrol are mediated by PKM2, our results provide an insight into molecular basis of resveratrol action. In MCF-7 cells, though resveratrol decreased PKM2 expression, the cancer metabolism was insignificantly altered. This observation correlated with earlier reports of lower aerobic glycolysis in non-invasive MCF-7 breast cancer cells [36]. Notably, in other highly invasive breast cancer cells like MDA-MB-231, high aerobic glycolysis confirms that our observation in MCF-7 is cell line specific and cannot be generalized for breast cancers [36]. Our results have also added a mechanistic insight into PKM2 expression down regulation by demonstrating that an inhibition of mTOR signaling is responsible for the process (Figure 2). Knock down studies indicated that PKM2 is critical for aerobic glycolysis and cellular proliferation (Figure 4) as well. These results are consistent with previous observations indicating that PKM2 is important for cancer cell glycolysis and growth [6], [12]. The observation of decreased intracellular R5P levels further extended the effect of resveratrol to inhibition of biosynthetic metabolism (anabolism). Essentially, we demonstrated that resveratrol inhibits metabolism of cancer cells, which in turn retards the cellular proliferation.

The decrease in PKM2 expression (Figure 1), following resveratrol treatment, provides resveratrol a therapeutic edge [6]. Additionally, the reversal of effects of resveratrol on PKM2 over-expression (Figure 7) implies that PKM2 is crucial in determining metabolic fate of cancer cells and is a critical target of resveratrol.

Our results have established previously unknown link between resveratrol and PKM2; thereby adding a new dimension to therapeutic potential of resveratrol. Results also endorse resveratrol as a promising anti-cancer agent in hindering pro-cancerous metabolism through PKM2 down regulation. Our observations suggest that resveratrol could be used in clinical trials for its contra effect on cancer metabolism via PKM2. Our results imply that natural compounds should be screened for their therapeutic potential and those known to possess anticancer properties should be investigated for their containing effects on cancer metabolism.
